# Behavioral Economics: “Nudging” Underserved Populations to Be Screened for Cancer

**DOI:** 10.5888/pcd12.140346

**Published:** 2015-01-15

**Authors:** Jason Q. Purnell, Tess Thompson, Matthew W. Kreuter, Timothy D. McBride

**Affiliations:** Author Affiliations: Tess Thompson, Matthew W. Kreuter, Timothy D. McBride, Washington University in St. Louis, St. Louis, Missouri.

## Abstract

Persistent disparities in cancer screening by race/ethnicity and socioeconomic status require innovative prevention tools and techniques. Behavioral economics provides tools to potentially reduce disparities by informing strategies and systems to increase prevention of breast, cervical, and colorectal cancers. With an emphasis on the predictable, but sometimes flawed, mental shortcuts (heuristics) people use to make decisions, behavioral economics offers insights that practitioners can use to enhance evidence-based cancer screening interventions that rely on judgments about the probability of developing and detecting cancer, decisions about competing screening options, and the optimal presentation of complex choices (choice architecture). In the area of judgment, we describe ways practitioners can use the availability and representativeness of heuristics and the tendency toward unrealistic optimism to increase perceptions of risk and highlight benefits of screening. We describe how several behavioral economic principles involved in decision-making can influence screening attitudes, including how framing and context effects can be manipulated to highlight personally salient features of cancer screening tests. Finally, we offer suggestions about ways practitioners can apply principles related to choice architecture to health care systems in which cancer screening takes place. These recommendations include the use of incentives to increase screening, introduction of default options, appropriate feedback throughout the decision-making and behavior completion process, and clear presentation of complex choices, particularly in the context of colorectal cancer screening. We conclude by noting gaps in knowledge and propose future research questions to guide this promising area of research and practice.

## Introduction

Low-income, minority, and uninsured people in the United States are disproportionately affected by illness and death from cancer and have lower rates of participation in recommended screenings (1,2). This article explores how insights from behavioral economics may reduce these disparities by providing tools to increase screening for breast, colorectal, and cervical cancer.

Breast, colorectal, and cervical cancer affect hundreds of thousands of Americans each year, and there is an evidence base for both supporting screening behavior and administering screening for all 3 of these cancer sites (3). The US Preventive Services Task Force (USPSTF) recommends that women receive screening mammography every 2 years from age 50 through 74 (3). For colorectal cancer, the USPSTF recommends screening for adults aged 50 to 75 using colonoscopy, sigmoidoscopy, or fecal occult blood testing (FOBT) (4). The availability of several screening methods with different recommended intervals adds to the complexity of a patient’s decision to be screened for colorectal cancer. For cervical cancer, the USPSTF recommends screening for women aged 21 to 65 by Papanicolaou (Pap) test every 3 years; women aged 30 to 65 may now choose a longer screening interval by combining cytology and testing for human papilloma virus (HPV) every 5 years (5). As part of Healthy People 2020, the US Department of Health and Human Services has set ambitious screening targets (compliance of 70.5% for colorectal cancer screening recommendations, 93.0% for cervical cancer, and 81.1% for breast cancer). However, underserved populations continue to be screened at lower rates (1,6).

Reaching the Healthy People 2020 goals in the face of persistent disparities requires new strategies for promoting screening tests. Three concepts from behavioral economics — judgment, decision-making, and choice architecture — can enhance existing evidence-based screening strategies by providing new insights into the behavior of patients and clinicians and suggesting new tools and techniques to promote screening.

## Behavioral Economics

We focus on 3 concepts from behavioral economics: judgment, decision-making, and choice architecture. The Table includes concepts in each area, with examples of how each can be applied to increase cancer screening in low-income and minority populations. Many of these ideas can be easily incorporated into strategies suggested by *The Guide to Community Preventive Services* (*Community Guide*) (7) (an evidence-based, accessible resource for health promotion and disease prevention, with specific coverage of breast, cervical, and colorectal cancer, compiled by an expert task force using systematic reviews as the basis for its recommendations), including evidence-based client- and provider-directed interventions to promote cancer screening, client reminders (eg, letters, post cards), small media (eg, brochures, newsletters), reducing structural barriers, provider assessment and feedback, one-on-one education, group education, and reducing client out-of-pocket costs (8).

**Table:** Selected Principles of Behavioral Economics and Examples of Approaches and Interventions for Breast, Cervical, and Colorectal Cancer Screening

Concept	Definition	Explanation of Concept	Application to Screening
**Judgment**			
Availability	People judge the likelihood of future events on the basis of how easy it is to imagine them or call up similar events in memory (32)	Highly memorable and positive messages about cancer screening will be easily recalled later when promoting screening. Personal stories, strong emotions, concrete and sensory language, and vivid imagery are memorable stimuli.	Use vivid language and personal stories to elicit strong emotion in communication about cancer screening to underserved populations.
Representativeness	People judge the probability or frequency of an event based on the extent to which it resembles similar past experiences they’ve had or assumptions they hold (9)	Highlighting similarities between the characteristics (eg, geographic, sex, racial, ethnic) of those who screen and the population targeted for increased screening may help motivate behavior change.	Present cancer screening as similar in a fundamental way to something that is already highly attractive or identifiable to the target population. If low-income men 50 or older value toughness, make screening something tough guys do.
Unrealistic optimism	People overestimate their personal success, ability, and immunity from risk compared with that of others (33,34)	Individuals may be overly optimistic about their *individual* chances of developing colorectal, breast, or cervical cancer and may fail to screen as a result.	Provide individual risk estimates (based on family history, lifestyle, sociodemographic group membership, etc) to encourage realistic estimates of risk.
**Decision-making**			
Affect	People evaluate options on the basis of basic emotional responses (liking/disliking, good/bad, approach/avoid) (30)	Eliciting strong positive affect in association with information about cancer screening should increase the likelihood that a person chooses to be screened. Countering expectations of negative affect (eg, fear, embarrassment) may have a similar effect.	Highlight the positive emotional aspects of screening (eg, relief, knowledge, agency) and provide reasonable counter arguments for, or forms of coping with, negative emotions (eg, fear, embarrassment, pain).
Context effects	When choosing among options, people are drawn to those that dominate all other options. If a dominant option is not clear, they tend to choose a compromise alternative with attribute values that lie between those of other options (32)	In the case of colorectal cancer, if one screening test dominated the others on some valued attribute (eg, cost, effectiveness, convenience), individuals would tend to choose it over other options, especially when given a choice that focused attention on that attribute.	Provide clear information about all pros and cons of various screening methods and help patients to choose the best option for their individual circumstances.
Discounting future rewards	People will make farsighted decisions when all costs and benefits occur in the future, but make shortsighted decisions when costs or benefits are immediate (32)	Many costs of cancer screening (eg, embarrassment, inconvenience, discomfort) are immediate, whereas many benefits (eg, prevention of cancer) are long term.	Reframe costs as minimal and identify immediate benefits. Provide an immediate incentive for cancer screening.
Fairness	People are more likely to leave a negotiation with nothing than to accept offers so low that they are perceived to be unfair (32)	Given mistrust of medical systems experienced by underserved populations, any perception of substandard treatment (eg, use of older technologies for screening) may result in failure to be screened.	Offer a full range of choices with information on what are considered “gold standard” options.
Framing effects	The way that choices are presented (ie, changing the relative salience of choice features) will often determine which options patients prefer (9,32)	Screening messages routinely emphasize that screening detects cancer early and prevents death or disease. But there are other ways to frame the choice to screen: “Would you prefer 80% to 90% certainty that you don’t have cancer or no certainty either way?” “Would you be willing to lose 20 to 30 years of your life in order to gain half-a-day without mild discomfort and inconvenience?”	Frame messages to encourage screening by noting both clinical effectiveness and the likelihood of additional benefits of being screened.
Social/cultural norms	People will observe what most of those in their peer group are doing and imitate their behavior (28)	If low-income adults perceive that others like them are not being screened for cancer (or don’t know anyone who has been screened), they will be less likely to be screened.	Provide evidence of social norms or stories from similar people who have been screened.
Loss aversion	When making choices under conditions of uncertainty, people prefer avoiding losses more than acquiring potential gains, even when the value and probability of both is the same (9,40)	Calling attention to what could be lost by choosing not to be screened for cancer (eg, valued life activities) along with potential gains of being tested (eg, peace of mind, sense of autonomy) may help increase screening.	Highlight the costs of failing to screen alongside benefits of screening.
**Choice architecture**			
Allowance for errors	A well-designed system expects its users to make mistakes and is as forgiving as possible when they do (33,34)	It should be easy to correct the “error” of not being screened.	Provide multiple reminders, on-demand scheduling and testing, and, in the case of colorectal cancer, the presentation of alternatives (eg, fecal occult blood test if you prefer not to undergo colonoscopy).
Default options	When given a choice with multiple options, including a pre-set default option, many people will choose (or accept) the default (33,34)	Health care providers could automatically schedule eligible patients for cancer screening at appropriate intervals (ie, a default option) with the understanding that patients who chose otherwise could opt out.	Provide automatic screening appointments as the default with the option to opt out.
Feedback	Telling people when they are doing well and when they are making mistakes improves performance (33,34)	Both positive feedback when screening has been completed and prompting feedback when screening opportunities have been missed or when screening is due should be integrated into systems of care for the underserved. This feedback may be more meaningful if presented alongside other health behaviors/risks.	Provide patients with a checklist of completed and uncompleted screenings at regular intervals.
Incentives	Behaviors are strongly influenced by the schedules of costs and rewards associated with them (33,34).	Monetary or other types of incentives that are relevant to the unscreened may be introduced to encourage cancer screening.	Provide incentives for completed screening.
Structuring complex choices	As choices grow in number, structure is necessary to increase the quality of decision-making (33,34)	Structuring choices about the types of cancer screening around such issues as timing, frequency, and invasiveness may increase understanding.	Give accessible explanations of screening choices and guide patients through what the typical experience for each entails.
Understanding consequences	The impact of competing options on welfare should be made clear (33,34)	People must be helped to understand the consequences of undergoing screening in terms of screening interval, screening process, ability to detect cancer, and follow-up treatment as needed.	Provide simple and meaningful information about the consequences of screening.

### Judgment

*Judgment* refers to the subjective assessment of the probability of outcomes or events. Decades of psychological and behavioral economics research has shown that judgments, particularly those in which people have limited experience or information, can be greatly influenced by seemingly irrelevant factors (9,10). Three aspects of judgment are relevant to cancer screening: availability, representativeness, and unrealistic optimism.

*Availability* refers to a tendency to judge the likelihood of future events, such as a cancer diagnosis, based on how easy it is to imagine them or call up similar events in memory (9,10). For example, women may be more likely to focus on their risk of breast cancer after the diagnosis of a family member or close friend because this experience is highly “available” in memory.

*Representativeness* refers to judgments about the probability or frequency of an event based on its resemblance to one’s past experiences or assumptions (9,10). If clinicians and health educators can provide memorable messages about cancer screening and show that people similar to the target audience undergo screening, then both availability and representativeness can be used to increase the perception of risk and highlight the benefits of screening.

*Unrealistic optimism* occurs when individuals have unreasonably low estimates of their own susceptibility to harm or overly high estimates of their chances of success or benefit (11). For example, people tend to underestimate their chances of receiving a diagnosis of cancer (12). Personally relevant messages that provide accurate information about cancer risk and the true prevalence of screening should increase uptake of screening tests, in part, by counteracting a tendency for unrealistic optimism.

### Decision-making

*Decision-making* examines the choices people make when faced with multiple options. The manner in which information is presented can influence decisions. *Framing effects* occur when the way choices are presented influences the options individuals prefer (13). In the classic example from early work in behavioral economics, people were presented with a scenario in which a disease is expected to kill 600 people and then given the choice of taking a course of action that would save 200 people or a course of action that had a one-third probability of saving 600 people and a two-thirds probability that no one would be saved. A majority chose the certain option of saving 200 people even though the probabilities are identical (13). Message framing promoting cancer screening also appears to affect the decision to be screened, particularly when messages are tailored with personal information (14). For example, women from underserved populations responded more favorably to letters from their primary care physicians that addressed their specific mammography beliefs, stage of change, perceived barriers, and risk factors than to a generic letter encouraging breast cancer screening. Several studies also suggest that framing the choice of colorectal screening tests should focus on the relative mortality risk reduction, sensitivity and specificity, preparation, and pain associated with each type of test (15,16).

Fairly consistent *context effects* also have been observed wherein people are frequently drawn to the one option in a set that dominates the others — or, if no single option dominates, the middle option between two extremes (17). For instance, colonoscopy often dominates the options for colorectal cancer screening among recommending physicians because of its effectiveness in detecting and preventing cancer, but compared with FOBT it may be seen as an extreme option among patients concerned about inconvenient preparation and invasiveness. This is an instance in which the context of the choices may differ depending on the perspective of an expert compared with that of a patient, which argues for shared decision-making and informed patient choice.

Health decisions also can be influenced by *loss aversion,* or a stronger preference for avoiding losses than acquiring gains (9,10). A consistent aversion to loss is a central finding in behavioral economics. Framing breast cancer screening messages in terms of loss aversion (eg, if a woman is not screened appropriately, she may risk loss of her health, her breasts, or her life) may have a minimal advantage over gain-framed messages (18). Loss-framed messages for diagnostic follow-up of abnormal cervical cancer screening are associated with heightened distress, particularly among women with attentional styles highly attuned to medical threats, but no difference in adherence compared with neutral or positive frames (19).

Messages about screening may also be framed to appeal to one’s sense of *fairness* (20). People who perceive the health care system as unfair (eg, have high levels of perceived discrimination or medical mistrust) are generally less trusting and less adherent to recommendations for preventive health behaviors such as cancer screening (21,22), though some exceptions to this finding have been noted (23). Addressing issues of trust and other barriers through improved communication, cultural competence, engagement in medical decision-making, and by providing better information regarding screening options may improve screening in underserved populations (24). Research suggests that people will often prefer to take nothing than to take an offer they believe is unfair (20). In the case of colorectal cancer screening, for example, there is a perception among some patients that a colonoscopy is a higher-quality procedure than an FOBT (25), which suggests that low-income and minority adults, especially those who mistrust medical providers or have experienced discrimination in past health care encounters, could reject screening altogether if the only test they were offered was one they perceived to be inferior to other options. A similar concern about fairness might be attached to mammography and prostate-specific antigen screening in light of recent USPSTF recommendations (26) and the burden of mortality among African Americans (27). Understanding individuals’ perceptions about the fairness of requiring (or recommending against) a particular test and their preferences for screening could help insurers communicate with specific subgroups about the appropriateness of various types of cancer screening.

Screening messages can also be framed in terms of *social and cultural norms* (28). Such messaging may describe the majority of a peer group engaging in behaviors such as screening. One study in men found that descriptive norms (ie, “what others like me do”) were predictive of cancer screening attitudes and intention, particularly when subjective norms (ie, “what significant others expect me to do”) were low. Those who received information that screening was common reported greater intention to be screened, rated their probability of being screened as higher, and were more likely to leave their names and addresses to get more information about screening (29). This finding has particular relevance for underserved populations, who may be unlikely to encounter frequent messages from family and friends regarding cancer screening.

*Affect*, or emotional response, can have a significant influence on decisions as well (30). Fear of cancer diagnosis, pain, and embarrassment have been recurring themes in the literature on barriers to cancer screening, particularly among the underserved (31). It may be effective to frame health messages to elicit positive affect about screening or counter negative affect regarding screening expectations. For example, describing the relief of knowing more about one’s health status or the sense of pride that comes with taking care of one’s health may be more effective than addressing the fear of a cancer diagnosis.

Finally, people tend to *discount future rewards*, or focus on immediate gratification rather than longer-term benefits (32). Therefore, it may be effective to reframe the costs of screening as minimal and identify immediate benefits, such as peace of mind or the ability to prevent cancer. Another strategy from behavioral economics that may offset future discounting is providing incentives to be screened. This allows screening to have an immediate and tangible benefit that may offset the inconvenience or discomfort of screening.

### Choice architecture

Synthesizing these principles, scholars in behavioral economics have proposed a set of strategies to counter some of the flaws in human judgment and decision-making in the face of uncertainty and risk. They suggest that optimal systems for high-stakes decisions will have 1) proper alignment of incentives, 2) an appreciation of how individuals understand the consequences of their decisions, 3) sensible default options, 4) appropriate feedback, 5) allowance for expected errors, and 6) a clear presentation of information for making complex choices (33,34).

*Choice architecture*, defined as “organizing the context in which people make decisions” (33,34), provides a promising means for encouraging screening. As competing choices grow in number, structuring complex choices can increase the quality of decision-making. As noted above, emphasizing aspects of colorectal cancer screening such as mortality risk reduction, specificity, and pain may help to organize options for patients. Default options exploit the tendency to accept the status quo when an option is presented as standard or prescribed. Defaults are powerful tools in promoting behaviors while respecting patient self-determination (35). Studies of interventions (ie, vaccination, HIV testing, organ donation) that use default options suggest that they may be a viable strategy to increase cancer screening (36). Substantial increases in participation across several behavioral domains are often observed by simply making the desired behavior the default option and requiring people to opt out (35), though the use of an opt-in strategy may be preferable for more controversial procedures such as HPV vaccination, where parents are more likely to consent when they can opt in (37). Providers could select 1 test and screening interval as the default and automatically schedule appointments for procedures while allowing patients to opt out.

Choice architecture also emphasizes feedback on performance. Information technologies that allow medical practices to track, remind, and provide feedback to both providers and patients are especially promising strategies for increasing cancer screening (24,38). Ideally, positive feedback should be provided when screening is completed, but prompts should also be given when screening opportunities are missed or when screening is overdue. Financial incentives to patients can also be effective in promoting screening. Incentives are among the most effective methods for increasing colorectal cancer screening in primary care (39). On the basis of promising findings in colorectal cancer screening and recommendations from the *Community Guide* regarding removal of structural barriers, incentives may be more appealing to low-income people if they could partially or fully cover costs incurred before screening (eg, transportation, time off of work, childcare) as opposed to being given in cash or noncash forms after screening. This may counteract the tendency to discount future rewards by adding proximal, immediate benefits of screening. Patients should also be assisted in understanding the consequences of not being screened and the consequences of various screening intervals and screening tests through informed decision-making that relies on both traditional presentations of risk and the principles of behavioral economics outlined above. Finally, a well-designed system should include an allowance for errors, making it easy for individuals to correct the “error” of not being screened with multiple reminders or on-demand scheduling and testing.

### Future directions for “nudging” underserved populations toward cancer screening

**Research.** Although we have noted some evidence supporting the principles of behavioral economics in cancer screening interventions, many research questions remain. There is some evidence that tailoring information to be consistent with patient characteristics and beliefs improves responsiveness to cancer screening recommendations, but fine-tuned experimental manipulations of behavioral economic factors such as availability, representativeness, affect, and social and cultural norms are needed as well. To test whether framing effects significantly alter attitudes and behaviors, randomized experiments should examine emotional reactions (eg, affect), information processing (eg, understanding), judgments and beliefs (eg, about cancer, about screening tests, about barriers), intentions (eg, to be screened), and actual screening behavior in response to various presentations of information. Research is also needed to test the speculation regarding fairness and the potential rejection of screening methods perceived as inferior.

Systems-level research should evaluate the effectiveness of organizing clinical practice to include elements of choice architecture, such as default options, feedback, incentives, and allowance for errors. In the area of incentives in particular, future research might also explore whether more invasive forms of screening require greater incentives among low-income adults, as might be expected based on research in other populations (15,16).

**Policy and practice.** In light of the promising evidence of effectiveness, changes in policy and practice may not need to wait on additional evidence to be implemented. For example, leading cancer organizations and federal agencies should consider advocating a “default option” approach to scheduling cancer screening appointments (with an opportunity to opt out) that is integrated into electronic medical records, particularly for providers and systems who serve underserved populations. Incentives delivered in ways that both facilitate (eg, transportation and child care) and reward (eg, gift cards) screening behavior should also be considered. At the very least, systems and providers should incorporate the recommendations made in this article and by others to include appropriate framing in their communications with patients regarding cancer screening decisions.

## Conclusion

Colorectal cancer, breast cancer, and cervical cancer are major causes of illness and death in the United States that disproportionately affect low-income and racial and ethnic minority populations. We have suggested a set of enhancements to existing strategies and related research questions to advance the use of behavioral economics in efforts to increase cancer screening in underserved populations. Many of our suggestions with respect to the framing of choices and message delivery could be added and evaluated within existing systems with little cost. Default options and provision of feedback are also consistent with the increasing use of clinical treatment guidelines and electronic medical records. The Affordable Care Act has also significantly lowered the cost of preventive treatments for underserved populations, but additional, creatively administered incentives may be necessary to translate increased access into completed screenings. The challenge of reducing, and ultimately eliminating, cancer-related health disparities is formidable, but tools from behavioral economics may move our efforts forward in meaningful ways by taking a realistic approach to human behavior and decision-making.
